# Supplementary material for: Genetic background modifies vulnerability to glaucoma-related phenotypes in Lmx1b mutant mice
Source: Dis Model Mech. 2021 Feb 19;14(2):dmm046953. doi: 10.1242/dmm.046953 (PMC7903917; doi:10.1242/dmm.046953)
Supplement: Supplementary information [file dmm-14-046953-s1.pdf]

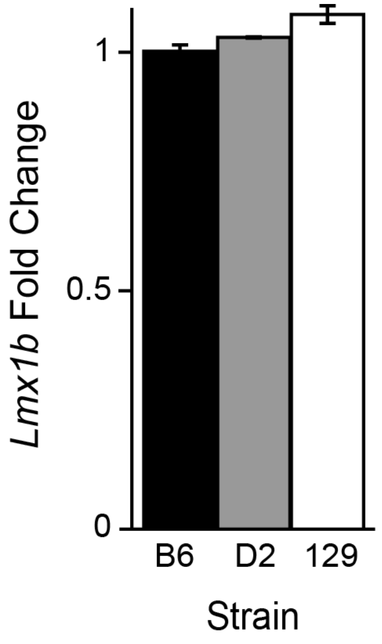

Figure S1: Ocular *Lmx1b* transcript levels are not different across tested inbred strains. *Lmx1b* expression in all anterior segment tissues (minus lens). There were no significant differences in *Lmx1b* transcript levels between the susceptible B6 strain and other backgrounds examined [Histogram ± S.E.M; fold change relative to B6 shown; n = 3 per group; B6 vs. D2, P = 0.641; B6 vs. 129, P = 0.093].

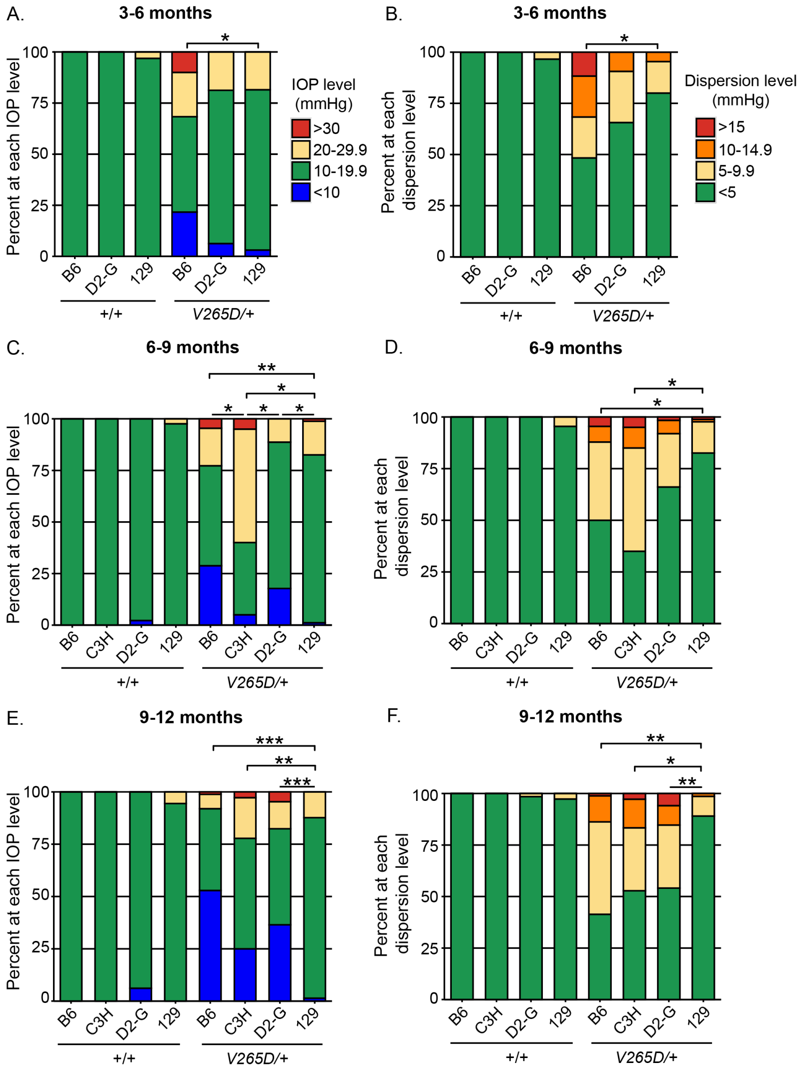

Figure S2: Frequency distributions of binned IOP values. The data shown in Figure 4 were binned to help to visualize strain difference. **A,C,E** Binned IOP values. **B,D,F** Binned IOP dispersion values. * P<0.01, ** P<1E-05, *** P<1E-10 (see supplementary table 2 for exact P-values).

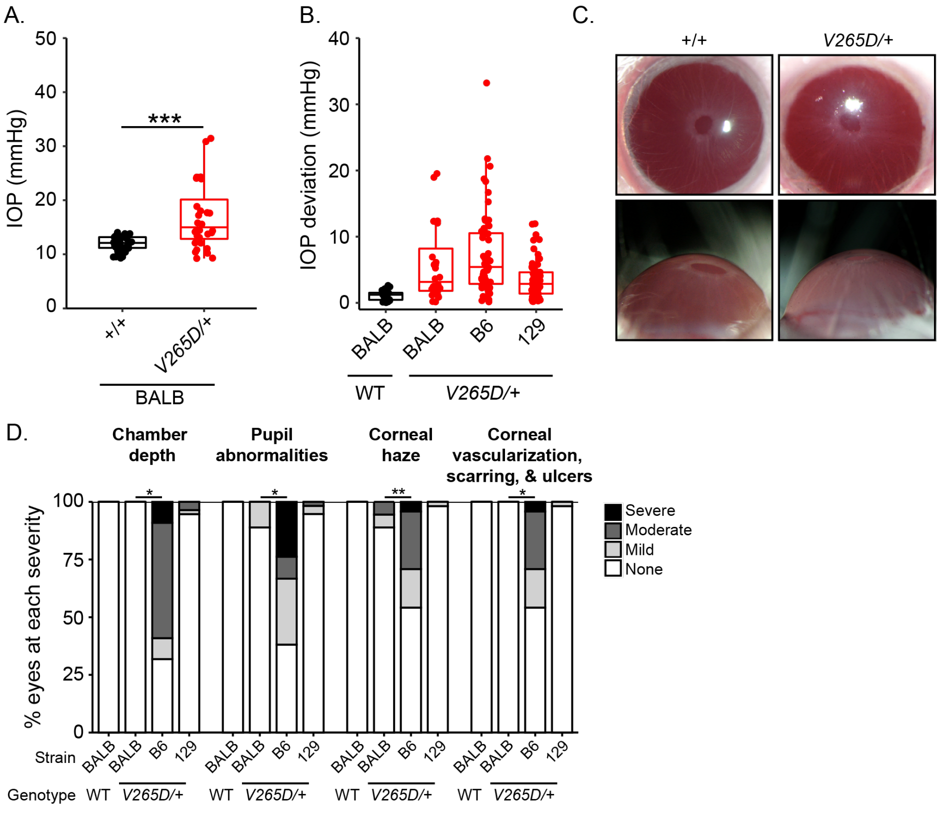

Figure S3: The tyrosinase deficient BALB background does not exacerbate *Lmx1b* mutant phenotypes. (A and B) Mutants on the BALB background have elevated IOP compared to WT controls (p = 5.4E-05). BALB background does not increase the IOP deviation compared to other pigmented backgrounds (B6 and 129). **(C)** Representative slit-lamp photos of both front and side views from BALB WT and mutant eyes at 3 months of age. **(D)** BALB mutant mice rarely have anterior segment abnormalities and when present they were mild. See supplementary table 2 for P values. All mice were examined between 3 and 6 months of age. At least 16 eyes were examined in each group. * P<0.01; ** P<1.0E-05; *** P<1.0E-10 (see supplementary Table 1 for P values).

Disease Models & Mechanisms • Supplementary information

Table S1:

A. Anterior chamber deepening *P* values

3-5 months old

Group	B6 WT (*n* = 24)	B6 HET (*n* = 22)	D2-G HET (*n* = 43)	129 HET (*n* = 56)	BALB HET (*n* = 18)
B6 WT	NA				
B6 HET	3.33E-07	NA			
D2-G HET	NA	0.0044	NA		
129 HET	NA	1.93E-08	2.76E-14	NA	
BALB HET	NA	1.92E-05	1.59E-07	NS	NA

6-8 months old

Group	B6 WT (*n* = 20)	B6 HET (*n* = 43)	C3H HET (*n* = 12)	D2-G HET (*n* = 48)	129 HET (*n* = 28)
B6 WT	NA				
B6 HET	2.32E-07	NA			
C3H HET	NA	NS	NA		
D2-G HET	NA	NS	NS	NA	
129 HET	NA	3.01E-08	0.00067	1.51E-10	NA

10-12 months old

Group	B6 WT (*n* = 40)	B6 HET (*n* = 68)	C3H HET (*n* = 42)	D2-G HET (*n* = 87)	129 HET (*n* = 31)
B6 WT	NA				
B6 HET	2.11E-16	NA			
C3H HET	NA	NS	NA		
D2-G HET	NA	NS	NS	NA	
129 HET	NA	1.08E-11	6.14E-18	2.26E-20	NA

B. Pupil abnormalities *P* values

3-5 months old

Group	B6 WT	B6 HET	D2-G HET	129 HET	BALB HET
B6 WT	NA				
B6 HET	4.71E-05	NA			
D2-G HET	NA	NS	NA		
129 HET	NA	2.83E-07	3.10E-04	NA	
BALB HET	NA	4.98E-03	NS	NS	NA

6-8 months old

Group	B6 WT	B6 HET	C3H HET	D2-G HET	129 HET
B6 WT	NA				
B6 HET	4.55E-09	NA			
C3H HET	NA	NS	NA		
D2-G HET	NA	NS	NS	NA	
129 HET	NA	1.22E-10	4.41E-07	2.83E-07	NA

10-12 months old

Group	B6 WT	B6 HET	C3H HET	D2-G HET	129 HET
B6 WT	NA				
B6 HET	3.03E-18	NA			
C3H HET	NA	NS	NA		
D2-G HET	NA	NS	NS	NA	
129 HET	NA	5.83E-12	7.26E-15	8.23E-10	NA

Disease Models & Mechanisms • Supplementary information

C. Corneal haze *P* values

3-5 months old

Group	B6 WT	B6 HET	D2-G HET	129 HET	BALB HET
B6 WT	NA				
B6 HET	6.20E-14	NA			
D2-G HET	NA	2.05E-10	NA		
129 HET	NA	6.78E-11	NS	NA	
BALB HET	NA	1.27E-09	7.61E-03	NS	NA

6-8 months old

Group	B6 WT	B6 HET	C3H HET	D2-G HET	129 HET
B6 WT	NA				
B6 HET	4.82E-15	NA			
C3H HET	NA	1.59E-05	NA		
D2-G HET	NA	1.97E-08	NS	NA	
129 HET	NA	2.42E-09	NS	NS	NA

10-12 months old

Group	B6 WT	B6 HET	C3H HET	D2-G HET	129 HET
B6 WT	NA				
B6 HET	2.69E-19	NA			
C3H HET	NA	NS	NA		
D2-G HET	NA	3.80E-06	0.00121	NA	
129 HET	NA	1.13E-06	0.00393	NS	NA

D. Corneal vascularization, scarring, and ulcers *P* values

3-5 months old

Group	B6 WT	B6 HET	D2-G HET	129 HET	BALB HET
B6 WT	NA				
B6 HET	2.21E-04	NA			
D2-G HET	NA	NS	NA		
129 HET	NA	1.61E-06	2.96E-05	NA	
BALB HET	NA	3.49E-03	7.41E-03	NS	NA

6-8 months old

Group	B6 WT	B6 HET	C3H HET	D2-G HET	129 HET
B6 WT	NA				
B6 HET	4.05E-07	NA			
C3H HET	NA	NS	NA		
D2-G HET	NA	NS	NS	NA	
129 HET	NA	9.72E-08	4.19E-06	1.15E-04	NA

10-12 months old

Group	B6 WT	B6 HET	C3H HET	D2-G HET	129 HET
B6 WT	NA				
B6 HET	1.55E-12	NA			
C3H HET	NA	NS	NA		
D2-G HET	NA	NS	NS	NA	
129 HET	NA	7.25E-11	1.49E-15	3.71E-16	NA

Disease Models & Mechanisms • Supplementary information

NA, Not applicable (to test); NS, not significant

Table S2:

A. IOP (Figure 4A-C) 3-6 months old

Strain Background	WT sample size	Mutant sample size	*P* value (WT vs mutant)
B6	55	60	NS
D2-G	32	32	2.20E-04
Strain 129	32	65	1.80E-05
BALB	36	32	5.44E-05

6-9 months old

Strain Background	WT sample size	Mutant sample size	*P* value (WT vs mutant)
B6	62	66	NS
C3H	13	20	0.0032
D2-G	45	62	0.0097
Strain 129	44	86	2.80E-04

10-12 months old

Strain Background	WT sample size	Mutant sample size	*P* value (WT vs mutant)
B6	61	87	8.20E-07
C3H	25	36	NS
D2-G	65	85	NS
Strain 129	37	73	NS

B. IOP deviation (Figure 4D-F) 3-6 months old *P* values

Group	B6 WT	D2-G WT	129 WT	BALB WT	B6 HET	D2-G HET	129 HET	BALB HET
B6 WT	NA							
D2-G WT	NS	NA						
129 WT	NS	NS	NA					
BALB WT	NS	NS	NS	NA				
B6 HET	3.50E-09	NA	NA	NA	NA			
D2-G HET	NA	3.10E-05	NA	NA	0.0068	NA		
129 HET	NA	NA	5.50E-06	NA	4.50E-05	NS	NA	
BALB HET	NA	NA	NA	4.16E-05	NS	NS	NS	NA

6-9 months old *P* values

Group	B6 WT	C3H WT	D2-G WT	129 WT	B6 HET	C3H HET	D2-G HET	129 HET
B6 WT	NA							
C3H WT	NS	NA						
D2-G WT	NS	NS	NA					
129 WT	NS	NS	NS	NA				
B6 HET	6.90E-11	NA	NA	NA	NA			
C3H HET	NA	0.0003	NA	NA	NS	NA		
D2-G HET	NA	NA	5.60E-08	NA	NS	NS	NA	
129 HET	NA	NA	NA	NS	1.00E-05	0.009	NS	NA

9-12 months old *P* values

Group	B6 WT	C3H WT	D2-G WT	129 WT	B6 HET	C3H HET	D2-G HET	129 HET
B6 WT	NA							
C3H WT	NS	NA						
D2-G WT	NS	NS	NA					

129 WT	NS	NS	NS	NA				
B6 HET	1.80E-17	NA	NA	NA	NA			
C3H HET	NA	8.8594E-07	NA	NA	NS	NA		
D2-G HET	NA	NA	7.4831E-12	NA	NS	NS	NA	
129 HET	NA	NA	NA	NS	3.0677E-11	2.4517E-05	5.6186E-08	NA

C. IOP distribution (Figure S1A,C,E)

3-6 months old *P* values

Group	B6 WT	D2-G WT	129 WT	B6 HET	D2-G HET	129 HET
B6 WT	NA					
D2-G WT	NS	NA				
129 WT	NS	NS	NA			
B6 HET	9.00E-12	NA	NA	NA		
D2-G HET	NA	6.60E-04	NA	NS	NA	
129 HET	NA	NA	NS	3.50E-05	NS	NA

6-9 months old *P* values

Group	B6 WT	C3H WT	D2-G WT	129 WT	B6 HET	C3H HET	D2-G HET	129 HET
B6 WT	NA							
C3H WT	NS	NA						
D2-G WT	NS	NS	NA					
129 WT	NS	NS	NS	NA				
B6 HET	1.50E-12	NA	NA	NA	NA			
C3H HET	NA	4.00E-04	NA	NA	NS	NA		
D2-G HET	NA	NA	6.60E-04	NA	NS	NS	NA	
129 HET	NA	NA	NA	NS	2.10E-07	1.10E-04	0.0012	NA

9-12 months old *P* values

Group	B6 WT	C3H WT	D2-G WT	129 WT	B6 HET	C3H HET	D2-G HET	129 HET
B6 WT	NA							
C3H WT	NS	NA						
D2-G WT	NS	NS	NA					
129 WT	NS	NS	NS	NA				
B6 HET	2.20E-16	NA	NA	NA	NA			
C3H HET	NA	0.000106	NA	NA	NS	NA		
D2-G HET	NA	NA	7.49E-10	NA	NS	NS	NA	
129 HET	NA	NA	NA	NS	1.80E-14	3.46E-05	6.65E-10	NA

D. IOP deviation distribution (Figure S1B,D,F)

3-6 months old *P* values

Group	B6 WT	D2-G WT	129 WT	B6 HET	D2-G HET	129 HET
B6 WT	NA					
D2-G WT	NS	NA				
129 WT	NS	NS	NA			
B6 HET	2.81E-11	NA	NA	NA		
D2-G HET	NA	3.57E-04	NA	NS	NA	
129 HET	NA	NA	NS	1.38E-04	NS	NA

6-9 months old *P* values

Disease Models & Mechanisms • Supplementary information

Group	B6 WT	C3H WT	D2-G WT	129 WT	B6 HET	C3H HET	D2-G HET	129 HET
B6 WT	NA							
C3H WT	NS	NA						
D2-G WT	NS	NS	NA					
129 WT	NS	NS	NS	NA				
B6 HET	5.56E-12	NA	NA	NA	NA			
C3H HET	NA	3.97E-04	NA	NA	NS	NA		
D2-G HET	NA	NA	1.13E-05	NA	NS	NS	NA	
129 HET	NA	NA	NA	NS	8.25E-05	5.08E-05	NS	NA

9-12 months old *P* values

Group	B6 WT	C3H WT	D2-G WT	129 WT	B6 HET	C3H HET	D2-G HET	129 HET
B6 WT	NA							
C3H WT	NS	NA						
D2-G WT	NS	NS	NA					
129 WT	NS	NS	NS	NA				
B6 HET	1.49E-15	NA	NA	NA	NA			
C3H HET	NA	9.41E-05	NA	NA	NS	NA		
D2-G HET	NA	NA	5.71E-10	NA	NS	NS	NA	
129 HET	NA	NA	NA	NS	1.06E-09	9.47E-05	6.20E-06	NA

NA, Not applicable (to test); NS, not significant

Disease Models & Mechanisms • Supplementary information

Table S3: Glaucoma GWAS genes on mouse Chromosome 18

Associated Gene	Mouse Chr. 18 Position (Mb)	Reference Paper	Phenotype Association
Arhgap12	60.2-61.3	(Craig et al., 2020)	Multitrait analysis of glaucoma
Cdc25c	34.7-34.8	(Craig et al., 2020)	Multitrait analysis of glaucoma
Jakmip2	43.5-43.7	(Craig et al., 2020)	Multitrait analysis of glaucoma
Mpp7	73.5-76.2	(Vishal et al., 2016)	Open angle glaucoma
*Tcf4**	69.3-69.7	(Choquet et al., 2017)	IOP elevation

*Lead SNP associated with both the *TCF4* and *LINC01415* loci
